# Natural Prey Preferences and Spatial Variability of Predation Pressure by *Cyphoma gibbosum* (Mollusca: Gastropoda) on Octocoral Communities off La Parguera, Puerto Rico

**DOI:** 10.1155/2014/742387

**Published:** 2014-10-28

**Authors:** Matthew Q. Lucas, Luis R. Rodríguez, Duane J. Sanabria, Ernesto Weil

**Affiliations:** Department of Marine Science, University of Puerto Rico, P.O. Box 9000, Mayaguez, PR 00680, USA

## Abstract

This study evaluated the natural prey preferences and spatial variability of predation pressure (PP = proportion of colonies with snails and/or clear predation signs) by the gastropod *Cyphoma gibbosum* on octocoral communities off the La Parguera Natural Reserve, Puerto Rico. All octocoral colonies were checked for presence of *C. gibbosum* and/or clear predation signs in four permanent band-transects (2 × 10 m), along three depth intervals (0–5, 7–12, >15 m deep) in each of six reefs along an inshore offshore gradient. Results indicate that *C. gibbosum* preys on at least 16 species, six of which (*Briareum asbestinum, Gorgonia ventalina, Pseudoterogorgia americana, P. acerosa, Plexaura flexuosa*, and *Pseudoplexaura porosa*) consistently showed significantly higher (K-W, *P* < 0.05) (17–37%) PP compared to all other species. *Plexaura flexuosa, P. americana*, and *P. porosa* had significantly higher PP (11–38%) among inner and mid-shelf reefs, and *G. ventalina* had higher PP in shelf-edge reefs (16–20%). A combination of differential spatial distributions and octocoral species abundances seems to explain the observed patterns of predation by *C. gibbosum*. Prey preference and higher abundances of 3-dimensional octocorals providing increased refuge or microhabitats utilized for mating or egg-deposition could be driving the spatial distribution of *C. gibbosum* and the observed differential predation pressure.

## 1. Introduction

Population outbreaks of coral predators are able to produce significant tissue/colony losses affecting overall productivity, population densities, and reef composition [1] and can be responsible for hindering reef growth by reducing their prey abundances and distribution [2]. Many predators inhabiting reef environments fully consume their small prey, but for large modular organisms such as corals and octocorals, partial mortality due to predation is more common. Their ability to recover from partial colony mortality has contributed to their ecological and evolutionary success [3].

Octocorals around the Caribbean thrive in shallow and intermediate depths (1–10 m), usually dominating habitats on exposed reef platforms [4]. They are subject to damage by storms and hurricanes and over the last decades, several species have suffered significant colony and/or partial mortalities due to outbreaks of diseases such as aspergillosis, black-band disease, red-band disease, bleaching, and other undescribed syndromes [5–13]. It seems that prevalence, virulence, and impact of these and other coral diseases have been increasing due to climate warming and bleaching events [14, 15]. Furthermore, increased predation pressure (PP) resulting in scarring or wounds to the colony may lead to infections by potential pathogens compromising the recovery and survivorship of the colony and the population dynamics in the community [2, 13].

The dominance of octocorals (Cnidaria: Octocorallia) in shallow Caribbean reefs has been partially attributed to their chemical defenses, which significantly limits predation impacts [16, 17]. As a result, there are only a few specialized predators known to intensely prey on octocorals [16]. The gastropod,* Cyphoma gibbosum* (Mollusca: Ovulidae), is the main predator of octocorals in the Caribbean and can be found to inhabit shallow water coral reefs in the wider Caribbean and southern Atlantic Ocean from North Carolina, USA, to Brazil [18–20]. A lack of intense predation on octocorals has been attributed to their ability to produce a diverse array of secondary metabolites or allelochemicals that deter predation by fish and other invertebrates [21]. Octocoral structural and chemical defenses, such as sclerite content [22, 23], and secondary metabolites (i.e., prostaglandins) [16, 24], have been investigated, but they do not support the observed patterns of prey preferences in* C. gibbosum*.

*Cyphoma gibbosum* is considered a trophic generalist because it is known to graze on numerous species representing at least three octocoral families [25] in spite of the toxic chemical defenses (allelochemicals) that they produce.* Cyphoma gibbosum* moves along the substrate from one octocoral colony to the next grazing mostly on the base and axial areas removing small quantities of live tissue and leaving the exposed endoskeleton and tissue scars. Despite the ability of* C. gibbosum* to heavily graze on octocorals, their impact has largely been considered modest since it often results in only partial mortality of the colony and tissues can quickly regenerate under normal conditions [26]. In spite of this, immune-compromised or physiologically weak octocoral colonies may not recover effectively, or they may become infected with progressive colony mortality [27]. Harvell and Suchanek [22] found that predation lesions by* C. gibbosum* vary from prey species to prey species, with superficial tissue damage in* B. asbestinum* and more drastic damage fully exposing the octocoral skeleton in* Plexaura* spp. Octocoral colonies normally harbor from one to three snails [25]; however, a population outbreak of* C. gibbosum* has been observed at Mona Island, where more than 150 snails were found grazing on large individual colonies resulting in total colony mortality [28].

A more recent study identified the ability of* C. gibbosum* to consume allelochemically rich octocoral prey may involve inducible biotransformation enzymes, such as cytochrome P450s (CYPs hereafter), that serve to detoxify allelochemicals defenses of octocorals [29]. On the other hand, predation on* C. gibbosum* is thought to be rare as most fishes find their mantle unpalatable and is generally ignored as a common prey item [30]. There are a few natural predators of* C. gibbosum*, such as the Caribbean spiny lobster (*Panulirus argus*), Hogfishes, (*Lachnolaimus maximus*) and Pufferfishes, Tetraodontidae spp. [30]. Groupers (Family Epinephelidae) have also been found to prey on* C. gibbosum* but only because they are indiscriminate feeders [31]. Harvell and Fenical [32] showed that the mantle provides protection through its distasteful or toxic qualities. This distastefulness or toxicity may arise from the sequestering of octocoral chemicals on which the snail feeds [18, 25]. A large-scale survey in the Florida Keys revealed that* C. gibbosum* abundances were greater in areas where large predatory fish were regularly harvested, concluding that the removal of top predators results in the release of their prey [33].

Previous studies have documented* C. gibbosum* grazing on a diverse array of octocoral species, mainly* Pseudoterogorgia* spp.,* Pseudoplexaura* spp.,* Plexaura homomalla,* and* G. ventalina* [25]. The spatial variability in the distributions of* C. gibbosum* foraging on octocorals has often been interpreted as feeding or prey preferences [25]. Likewise,* C. gibbosum* may select its octocoral prey for other than just food sources; they could be using it as refuge from predation and sites for mating and/or egg deposition [18]. Previous research exploring prey preferences of* C. gibbosum* on octocoral communities has yielded disparate results and various authors report different octocoral species as the preferred prey. Kinzie [34] found no preferences and related prey utilization to octocoral species abundances, whereas Birkeland and Gregory [35] reported* Gorgonia* spp. and* Eunicea succinea* as preferred prey. At Salt River Canyon, St. Croix USVI, Harvell and Suchanek [22] identified various species of* Plexaura* harboring snails for longer periods than expected and suggest that feeding preferences alone do not explain the foraging patterns of* C. gibbosum.* Instead, the authors partition the foraging patterns of* C. gibbosum* to various social behaviors (e.g., mating, egg deposition, feeding, and predator avoidance) [22]. In Panama,* Pseudoterogorgia* spp.,* P. homomalla*, and* P. porosa* were found to be the preferred prey [25]. This variability in food preferences seems to reflect sampling variation, temporal and geographical differences in preferences, and/or prey quality [25]. Furthermore, different behaviors can lead to misinterpretation of octocoral species as preferred prey including host species selection, residence time on the host, mating, egg deposition, and grazing rates [25]. The authors conclude that snail social interactions and predator avoidance behaviors play an important role in* C. gibbosum's* prey preferences, and rather than referring to octocorals as prey they may be best described as hosts [25], serving a variety of the aforementioned ecological roles.

Predation by* C. gibbosum* on octocoral communities has never been assessed among the reefs of the La Parguera Natural Reserve, located off the southwest coast of Puerto Rico. Populations of most natural predators of* C. gibbosum* in this area have been overfished (reviewed in [36]), resulting in reduced predation pressure on this predatory snail. Coral and octocoral populations have been affected by several bleaching events and disease outbreaks over the last 20 years and a significant increase in octocoral predation signs by* C. gibbosum* has been observed [14] (E. Weil pers. obs.) underpinning the potential impact of increased predation pressure of this snail over several species of common octocorals. The* C. gibbosum* population outbreak with high colony and tissue mortalities reported from the waters of Mona Island [28] serve as an example of the potential impact of this snail on local octocoral communities in the waters of the La Parguera Natural Reserve and other Caribbean localities. Therefore, it is important to assess the current status of populations of* C. gibbosum* and its common prey species. The objectives of this study were to (1) identify the natural prey preferences of* C. gibbosum* and (2) characterize the spatial variability of predation pressure by* C. gibbosum* on octocorals among La Parguera reefs. Furthermore, we address the potential impact of predation pressure on different octocoral species and the community. The results are discussed in light of previous research and the ecological factors potentially contributing to the natural prey preferences and the spatial variability of predation pressure by* C. gibbosum* on the octocoral communities of southwestern Puerto Rico.

## 2. Materials and Method

### 2.1. Study Location

**Figure 1:**
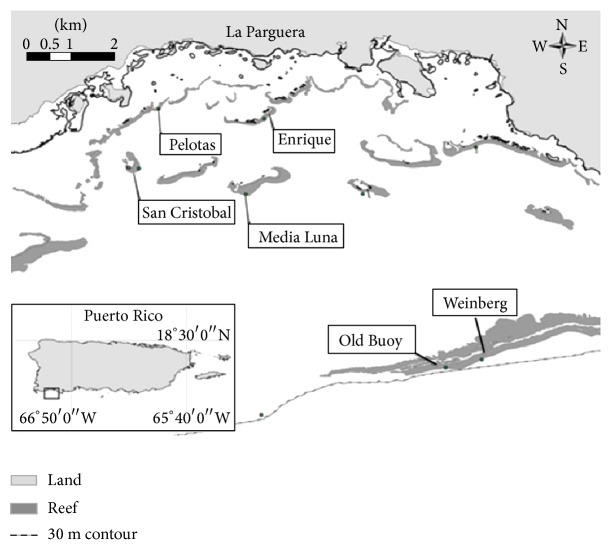
A map of the La Parguera Natural Reserve located off the southwest coast of Puerto Rico showing the location of the six reefs sites surveyed in this study.

Coral reefs of the La Parguera Natural Reserve are dispersed over an insular shelf extending 8–10 km offshore [36] consisting of three distinct inshore-offshore zones: (1) the inner reef zone that is mainly formed by shoreline fringing mangrove forests as well as fringing and patch reefs close to shore, (2) the mid-shelf zone characterized by reefs fringing along coral rubble and mangrove keys oriented from east to west at about 2-3 km from the coast, and (3) the shelf-edge zone which is characterized by deep spur and groove and bank reef formations [37]. For this study two inner reefs, Pelotas (17°57.442 N, 67°04.176 W) and Enrique (17°56.658 N, 67°02.213 W), two mid-shelf reefs, Media Luna (17°56.093 N, 67°02.931 W) and San Cristobal (17°56.501 N, 67°04.509 W), and two shelf-edge reefs; Weinberg (17°53.429 N, 66°59.320 W); and Old Buoy (17°53.380 N, 66°59.090 W) were selected (Figure 1). At each reef site, all octocoral colonies were checked for presence of* C. gibbosum* and/or predation signs in each of four haphazardly placed, permanent 20 m^2^ band transects (10 × 2 m) in each of three depth habitats (3–5, 5–10, and >15 m) in the inner- and mid-shelf reefs. At the shelf-edge reefs, the 12 band-transects were distributed at depths between 18 and 25 m.

Predation pressure (PP hereafter) is herein defined as the proportion (%) of octocoral colonies harboring snails with signs of predation and/or octocorals with only clear and recent predation signs. Data collected included the species or genus of the octocoral colony with snails and/or predation signs, the number of snails on each colony, and the total number of octocorals within each band-transect. The proportions of snails per octocoral colony were estimated for each species and for the octocoral community. In addition, the mean proportion (%) (±standard deviation or standard error) of colonies with predation signs and/or snails (PP) was estimated for each species in each habitat, within each reef, and zone. Octocoral species with no predation signs and/or with low population abundance were not included in downstream analyses (Table 1).

### 2.2. Data Analyses

#### 2.2.1. Natural Prey Preferences of* C. gibbosum*

The occupancy patterns of* C. gibbosum* were evaluated to determine if the spatial variability in PP by* C. gibbosum* was related to the abundances (octocoral spp. density) of preferred octocoral species. Ivlev's index of electivity was used to estimate natural prey preferences of* C. gibbosum* on octocoral species [33, 38]. Prey electivity (*e*) = (*r*_*i*_ − *P*_*i*_)/(*r*_*i*_ + *P*_*i*_), was estimated whereby *r*_*i*_ is the proportion of prey species *i* utilized and *P*_*i*_ is the proportion of prey species *i* available. Ivlev's index rates species utilization from −1 to +1, with −1 indicating total rejection, 0 indicating that prey is taken in proportion to their abundances, and +1 indicating a preference of host species to the exclusion of others [33, 39]. Octocoral species abundances (densities) were estimated for each transect, habitat (depth), and reef by pooling the data. Spearman rank correlation analyses were performed to assess the relationship between octocoral abundances (density) and PP (*α* = 0.05).

#### 2.2.2. Spatial Variability of Predation Pressure (PP)

To test the hypothesis that there is no spatial variability in* C. gibbosum* and octocoral population densities, data was tested for normality and equality of variance with the Shapiro-Wilk test, followed by a one-way ANOVA or Kruskal-Wallis (K-W) tests. To test the hypothesis that PP by* C. gibbosum* on the main octocoral species is similar among habitats within reefs, between reefs within zones, across reefs and reef zones, the mean proportion (%) values for PP for each spatial level (habitat, reefs, and zones) were calculated, arcsine transformed, and independently compared using one-way ANOVA or K-W tests. If significant differences were found, pairwise differences were tested with Tukey test. Statistical analyses were performed (*α* = 0.05) using SigmaStat v.10.0 (Systat Software, San Jose, CA).

## 3. Results

### 3.1. Natural Prey Preferences of* C. gibbosum*

A total of 219 snails were found grazing on 16 different octocoral species spanning eight genera and four families (Briaridae, Plexauridae, Gorgonidae, and Anthothelidae). Of the 16 species,* B. asbestinum* (12.3%),* P. americana* (7.3%),* P. flexuosa* (7.1%),* G. ventalina* (5.8%),* P. porosa* (4.7%), and* P. acerosa* (1.5%) were the most frequently observed octocoral species harboring snails (Table 1). However, octocoral species with low abundances and colonies harboring snails outside of the experimental band-transects were not considered in downstream analyses (see Table 1).

Octocoral species densities were relatively uniform from inshore to offshore reefs (Table 2). Ivlev's prey electivity index rating [39] indicates that* C. gibbosum* preys on various octocoral species relative to their abundance or availability (Table 3). Spearman rank correlations (*Rs* coefficient) between octocoral occupancy (colonies with snails) and octocoral availability (palatable prey) covaried significantly at inner (*Rs* = 0.94, *P* < 0.05, *P* = 0.006) and shelf-edge reefs (*Rs* = 0.68, *P* < 0.05, *P* = 0.003), suggesting that as palatable octocoral availability increases, snail occupancy also increases (Table 3). In contrast, there was no significant correlation for mid-shelf reefs, which had the fewest snails (*n* = 40) and the highest number of octocorals surveyed (*n* = 1421 colonies) of all reefs (Table 3).

Spearman rank correlations between mean (%) PP and densities of each octocoral species showed no significant covariation for most species (Table 4). However, there was significant (*Rs* = 0.09, *P* < 0.05, *P* = 0.02) positive covariation between mean (%) PP and octocoral density for* P. porosa* and significant negative (*Rs* = −0.94, *P* < 0.05, *P* = 0.02) covariation for* G. ventalina* (Table 4). No significant correlations were found between mean (%) PP and the pooled densities for all octocoral species within each reef (Table 5). These results indicate that along an inshore-offshore gradient,* C. gibbosum* largely preys on six different octocoral species relative to the proportion of their abundance in the communities off La Parguera, Puerto Rico.

### 3.2. Spatial Variability of Predation Pressure (PP) by* C. gibbosum*

**Figure 2:**
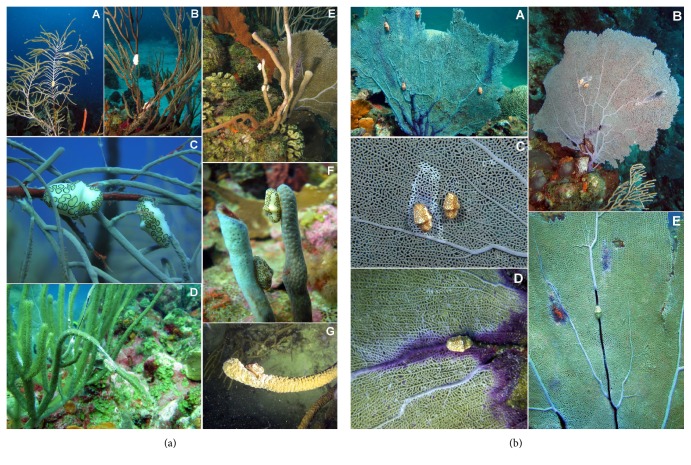
(a) Photographs showing the snail* Cyphoma gibbosum* grazing on several species of octocoral. Two snails each on a single colony of* P. americana*,* P. homomalla,* and* P. acerosa* (A, B, C). A branch of* P. porosa* with a large portion of tissue eaten by one snail (D). Snail eating tissue from* B. asbestinum* (E, F) and* P. nutans* (G). (Photo credit: E. Weil). (b) Photographs showing a medium size colony of* G. ventalina* with four snails and scar areas produced by its feeding activity on the main axis and blade (A, B). Close-up of the damage produced by the snail feeding (C, D) and a large colony showing the impact of predation activity of one snail along the main axes and the blade (E). These damaged tissue areas are susceptible to infections by pathogens and/or could be rapidly colonized by algae, sponges, or* Millepora* which could increase sea fan tissue mortality (Photo credit: E. Weil).

**Figure 3:**
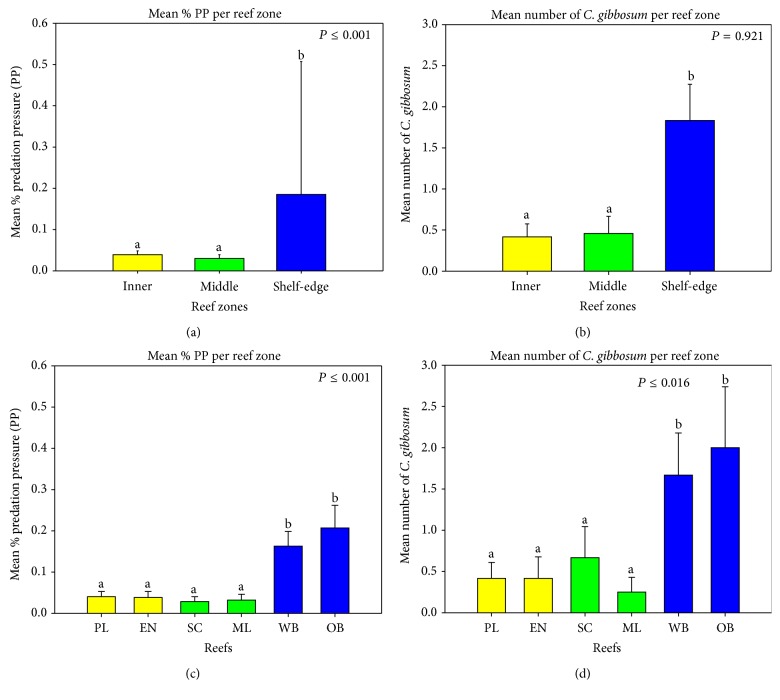
Mean (%) predation pressure by* C. gibbosum* on the octocoral community (pooled data) for each reef zone (upper left) and reef locality (bottom left) and their associated mean* C. gibbosum* densities (right column).* Reef Sites*: PL= Pelotas; EN= Enrique; SC= San Cristobal; ML= Media Luna; OB= Old Buoy; WB= Weinberg.

Snail densities reached 0.15 ind./m^2^ among 3,589 octocoral colonies (2.5 ind./m^2^) within the 72 band-transects surveyed in this study (Table 1). Overall, six octocoral species (*Briareum asbestinum*,* Pseudoterogorgia americana*,* Plexaura flexuosa*,* Gorgonia ventalina*,* Pseudoterogorgia acerosa,* and* Pseudoplexaura porosa*) were the most affected by snail predation (Table 6, Figure 2(a)). Snails were most often found at the base of the colonies or along the main axis, with few along the branches of stiliform, plume-like, or candelabra-like growth forms. For the species* G. ventalina*, snails were generally found at the base and along the main axes with fewer snails eating on the open fan structure (Figure 2(b)). When pooling all octocorals sampled, no significant differences in* C. gibbosum* densities and/or PP were found among depth habitats within reefs (data not shown). Similarly, no significant differences in snail densities and/or PP were found between reefs within each zone (data not shown). In contrast, the shelf-edge zone had significantly higher (K-W, *P* < 0.05, *P* = 0.001) snail densities and PP compared to the inner- and mid-shelf reefs (Table 6, Figure 3).

**Figure 4:**
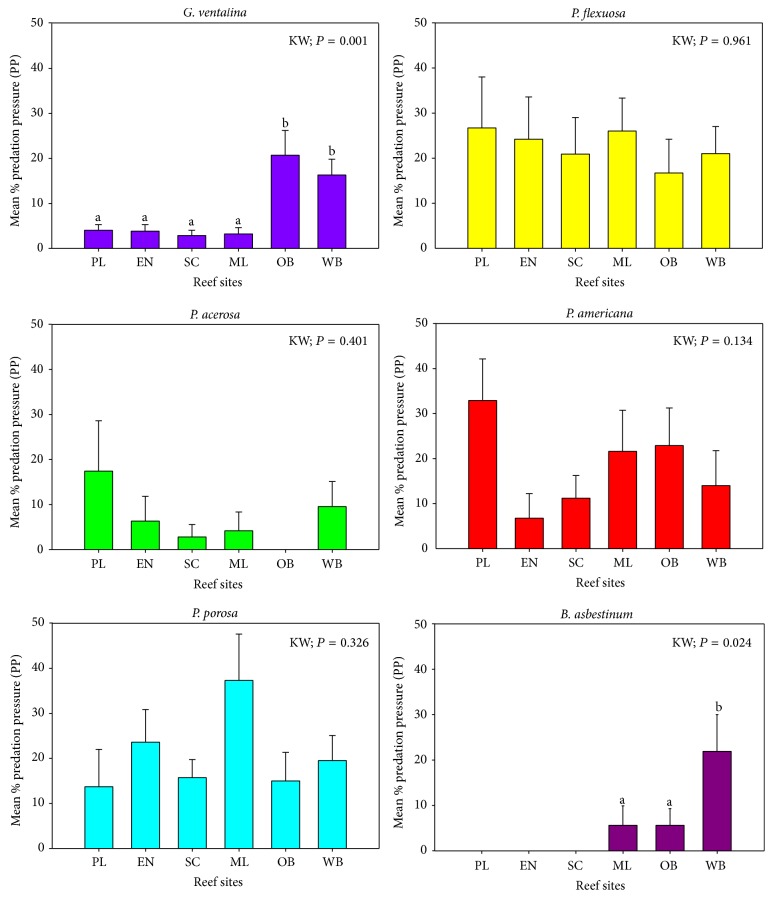
Mean (%) predation pressure variability by* C. gibbosum* on the most affected octocoral species across reefs in La Parguera. Error bars represent the standard error.* Reef Sites*: PL = Pelotas; EN = Enrique; SC = San Cristobal; ML = Media Luna; OB = Old Buoy; WB = Weinberg.

In general, there was high variability in PP within each of the preferred species across reefs.* Briareum asbestinum* and* G. ventalina* were the only species with significant differences in PP across reefs.* Briareum asbestinum* had significantly (K-W, *P* < 0.05, *P* = 0.024) higher PP at Weinberg (21.9%) compared to the other five reefs, while* G. ventalina* had significantly (K-W, *P* < 0.05, *P* = 0.001) higher PP at Weinberg and the Old Buoy (16–20%) compared to reefs in the inner- and mid-shelf zones (2–4%) (Table 6, Figure 4).* Pseudoterogorgia americana* had the highest PP in Pelotas (32.9%) compared to Enrique (lowest) (6.7%), and PP was similar in all the other reefs (Table 6, Figure 4).* Pseudoplexaura porosa* (13.7–37.3%),* P. flexuosa* (16–26.7%), and* P. acerosa* (2.8–17.4%) showed high, but not significant, variability in PP across reefs (Table 6, Figure 4). This lack of statistical significance is mainly due to the high variance around the means produced by differential levels of PP in the sampling units (i.e., some reefs had transects with few or no colonies with PP, while others had numerous colonies with high PP).

**Figure 5:**
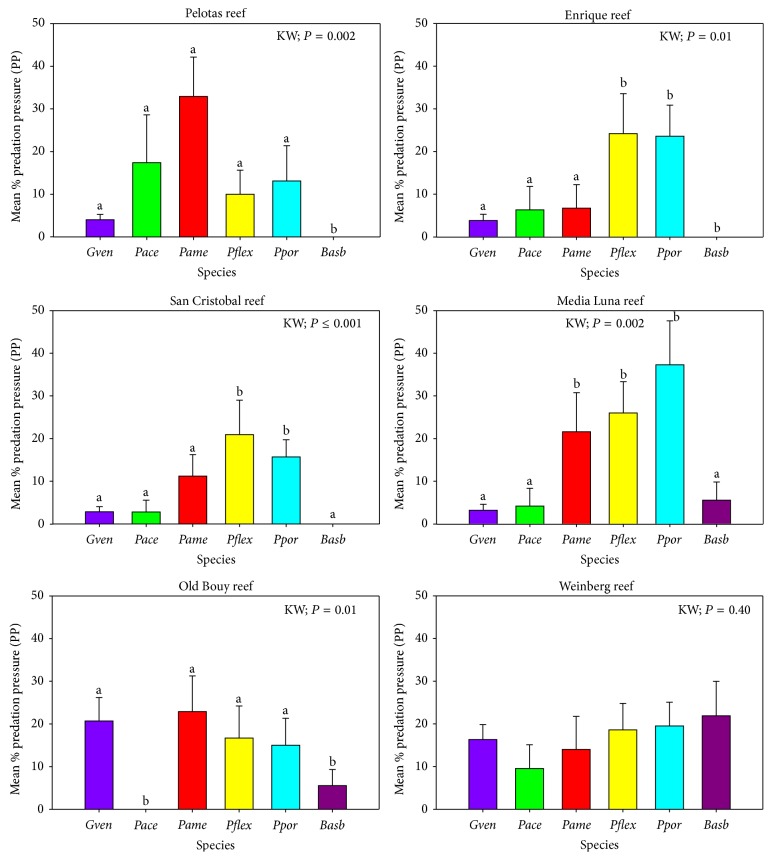
Variability in mean (%) PP by* C. gibbosum* across the six major octocoral prey species within each reef off La Parguera. Error bars represent the standard error.

No species-specific patterns were observed when comparing PP among species within reefs (Table 6, Figure 5).* Briareum asbestinum* had significantly (K-W, *P* < 0.05) lower PP (5.6%) in the Old Buoy compared to* P. flexuosa*,* P. americana,* and* P. porosa* in Media Luna (Table 6, Figure 5).* Plexaura flexuosa* and* P. porosa* had significantly (K-W, *P* < 0.05) higher PP compared to* G. ventalina*,* P. acerosa*, and* B. asbestinum* in Enrique, Media Luna, and San Cristobal, but they were similar to the other species in the two shelf-edge reefs (Table 6, Figure 5).* Pseudoterogorgia americana* had significantly higher (K-W, *P* < 0.05) PP compared to* G. ventalina* and* B*.* asbestinum* in Pelotas and Media Luna reefs.* Pseudoplexaura porosa* and* P. flexuosa* had significantly higher (K-W, *P* < 0.05) PP than* G. ventalina* and* P. acerosa* in the mid-shelf reefs and no significant differences in PP were found across species in both shelf-edge reefs (Table 6, Figure 5).

## 4. Discussion

This study found no consistent patterns in the levels of PP across the inshore-offshore gradient for all octocoral species surveyed. Nonetheless, the sea fan* G. ventalina* showed the highest PP in the two offshore reefs where its own densities and that of the other branching and plume-like species were lower than in mid- and inner-shelf reefs. However,* G ventalina* was the most abundant octocoral (accounting for 40%) of all octocorals surveyed in the spur and groove formations of the shelf-edge zone.

Predation on* C. gibbosum* by fish and other invertebrates has been proposed as one of the mechanisms controlling its abundances and distribution [30, 33] and therefore may be influencing the differential distribution of* C. gibbosum's* PP on octocorals. The high abundances of snails on sea fans in the two offshore reefs may indicate lack of significant PP on the snail due to overfishing in combination with low densities of the snail's other preferred octocoral prey. Results for the other reefs suggest that if* G. ventalina* and the other preferred species have similar and/or high densities,* C. gibbosum* prefers them over* G. ventalina*, which showed significantly lower PP in these reefs. This raises the question as to whether prey preference is driven by the quality of the food supply, the refuge provided by the structurally more complex plume and branching species, or other ecological functions, such as mating or egg deposition sites that the octocoral host provides the snail [25] and how it is regulated by natural predation pressure on the snail. Burkepile and Hay [30] investigated the impact of snail predators using cage and uncaged treatments and showed that, when large predators were excluded, there were significant increases in snail densities and predation on octocorals. The removal of large predators allowed an increase of* C. gibbosum* abundances (19x) consequently resulting in an increase in predation on octocorals. They further stated that a (0.8 meter) tall colony of* Eunicea calyculata* displayed approximately 75% live tissue mortality [30].

While the impact of* C. gibbosum* is considered minimal, given the intense fishing pressures and the rapid removal of their predators, there is precedent for concern of outbreaks negatively affecting octocoral communities. Reefs in La Parguera continue to be heavily overfished with significant changes in species composition and fish community structure in coral reefs [15, 36], which may explain the observed increase in population densities of the snail in the area over the years (E. Weil pers. obs.). This (overfishing) in combination with successful reproductive events could increase the likelihood of snail population outbreaks, such as the one observed at Mona Island in 2010 [28], which could lead to significant increase in PP on the octocoral community of La Parguera's reefs. A similar condition has been observed in another coral predator by the gastropod* Coralliophila abbreviata*, which has a wide list of scleractinian coral prey [39–41]. Bruckner et al. [41] followed the movements and feeding rates of* C. abbreviata* on* Acropora palmata* off La Parguera. Their findings showed that a combination of reduced* A. palmata* densities due to the white band disease epizootic, environmental disturbances, and overfishing of natural predators of the snail could increase abundances and significant predation damage by* C. abbreviata*, contributing to the demise of* A. palmata* colonies inhabiting the inshore reefs of southwestern Puerto Rico [41]. Similarly, a 75% decrease in* A. palmata* colonies in the Florida Keys occurred after Hurricane Georges (1998), followed by doubling of the proportion of colonies infested with snails (from 19% to 46%) and increase in snail density per infested colony as snails concentrated on surviving* A. palmata,* which significantly increased the predation impact on* A. palmata* populations [42].

Overall, the spatial variability of PP and Ivlev's index of electivity suggests that* C. gibbosum* naturally preys on* B. asbestinum*,* P. americana*,* P. acerosa*,* P. flexuosa*,* G. ventalina*, and* P. porosa* relative to their abundances or availability across reefs. Although, Ivlev's index weights electivity for rare species disproportionately, the combined results of PP and electivity in this study corroborate the notion that prey preference seems to be related to octocoral abundances rather than feeding preferences alone. In addition, correlation analyses between mean (%) PP and the density of individual octocoral species within each reef revealed no significant covariation for most species, however. In contrast, there was a weak negative correlation between the mean (%) PP and the densities of* G. ventalina* colonies across all reefs. Similarly, there was a strong positive correlation between the mean (%) PP and the densities of* P. porosa* across all reefs.

These data indicate that PP by* C. gibbosum* increases as octocoral abundances decrease. The result corresponds with the observation that* C. gibbosum* differentially preys or utilizes different octocoral species relative to their abundances. For example, when stiliform and plume-like, or candelabra-like growth forms are abundant and readily available they are preyed on more frequently than the openly exposed fans of* G. ventalina*, which also renders them vulnerable to predation by fish, lobsters, and so forth. In addition, there were no significant correlations between the mean (%) PP and the pooled snail densities for all octocoral species within each reef, thus supporting the notion that, when octocoral species abundances vary, predation by* C. gibbosum* will vary accordingly. In general, these results suggest that the natural prey preferences of* C. gibbosum* might be linked to a combination of differential spatial distributions and octocoral species abundances, feeding preferences of* C. gibbosum*, and predation pressure on snail populations.

Chiappone et al. [33] suggested for spur and groove reefs in Florida that most octocoral hosts are readily available and are probably not food limited due to* C. gibbosum's* ability to tolerate high levels of octocoral chemical defenses [43]. Whalen et al. [29] investigated the genetic diversity, transcriptional responses, and enzymatic activities of cytochrome P450s (CYPs) in seven octocoral species potentially linked to detoxification of allelochemicals in* C. gibbosum*. The authors link the induction of specific CYP transcript expression and corresponding enzymatic activity in* C. gibbosum* to differences in octocoral prey [29]. Specifically, their results show that only snails consuming* P. homomalla* demonstrated greater induction of CYP transcripts (2.7- to 5.1-fold) and a corresponding increases in the metabolic activity of eicosanoid LTB4 (i.e., a prostaglandin that serves as a feeding deterrent molecule) in the digestive gland of snails [29]. Their findings are consistent with previous research indicating that the tissues of the Caribbean genus* Plexaura* contain high concentrations of prostaglandins [29, 44]. The authors conclude the possibility that allelochemicals in* P. homomalla* induce* Cyphoma* CYP4 enzymes and may serve to detoxify chemical defenses of octocorals. Although their findings are no indication of prey preferences in* C. gibbosum*, predation on* Plexaura* species rich in unique prostaglandins may serve as an evolutionary innovation in* C. gibbosum's* ability to tolerate allelochemically rich octocoral prey [29], as well as securing prey that is not readily available to other predators. Future* in-situ* and laboratory experiments may provide better insight on more specific prey preferences and increase our understanding of the feeding behavior, ecological dynamics, and the potential of* C. gibbosum* to regulate octocoral community structure in Caribbean reef communities.

## 5. Conclusions

The predatory snail* C. gibbosum* was observed on 16 different octocoral species spanning eight genera and four families (Briaridae, Plexauridae, Gorgonidae, and Anthothelidae). Although this study lacks temporal observations, the spatial distribution (differential abundances) of preferred octocoral species, local ecological conditions, and reduced snail predation pressure are the most parsimonious factors explaining predation patterns of* C. gibbosum* on the octocoral communities off the coast of La Parguera. The predatory or foraging behavior of* C. gibbosum* is not likely to have detrimental impact on octocoral populations under low snail population densities and high prey densities; however, population outbreaks of the snail or high mortalities of preferred prey or a combination of these are bound to significantly increase the impact of predation by the snail on octocoral communities. In La Parguera, overfishing has removed most snail predators and there is concern over population outbreaks of invertebrate corallivorous species, such as* C. gibbosum* and* C. abbreviata*, resulting in increased predation to octocoral and coral colonies, loss of reproductive tissue, and a source of vectoring diseases. Future work should include long-term laboratory and* in situ* studies that investigate the partitioning of ecological behaviors, including mating, egg deposition, and foraging patterns and prey preferences across octocoral communities. Monitoring octocoral communities and their predators will be increasingly important in broadening our understanding of the complex trophic interactions among Caribbean coral reef communities.

## Figures and Tables

**Table 1:** Total octocoral species and the number of colonies harboring snails. The total number of band-transects (20 m^2^) surveyed in the entire study was 72.

Octocoral species	Total colonies	Number of snails	% Snails
*Briareum asbestinum *	130	16	12.3%
*Pseudoterogorgia americana *	827	61	7.3%
*Plexaura flexuosa *	532	34	6.3%
*Gorgonia ventalina *	1153	67	5.8%
*Pseudoplexaura porosa *	425	21	4.9%
*Plexaura homomalla *	90	4	4.4%
*Plexaurella nutans *	30	1	3.3%
*Pseudoterogorgia acerosa *	397	6	1.5%
*Pterogorgia guadalupensis *	2	1	—
*Muricea* spp.	1	1	—
*Eunicea* spp.	2	2	—
^*^ *Erythropodium caribaeorum *	1	1	—
^*^ *Pterogorgia anceps *	1	1	—
^*^ *Pterogorgia citrina *	1	1	—
^*^ *Gorgonia flabellum *	1	1	
^*^ *Gorgonia mariae *	3	1	—

^*^Low abundant octocoral species and colonies observed with snails outside of band-transects were not considered in downstream analyses.

**Table 2:** Octocoral species densities (colonies/m^2^) for the six most affected octocoral species within each reef along an inshore to offshore gradient.

Reef Zone	Inner-shelf reef		Mid-shelf reef		Shelf-edge reef	
	Pelotas	Enrique	San Cristobal	Media Luna	Old Buoy	Weinberg
Octocoral spp.						
* B. asbestinum *	0.07	0.09	0.05	0.19	0.04	0.15
* G. ventalina *	0.73	0.81	1.10	0.86	0.63	0.67
* P. americana *	0.94	0.98	0.65	0.42	0.18	0.13
* P. acerosa *	0.24	0.35	0.32	0.49	0.03	0.18
* P. flexuosa *	0.10	0.55	0.58	0.46	0.20	0.33
* P. porosa *	0.10	0.32	0.32	0.44	0.26	0.33

**Table 3:** Ivlev's prey electivity index for each reef zone for octocoral species harboring snails. Prey electivity (*e*) = (*r*_*i*_ − *P*_*i*_)/(*r*_*i*_ + *P*_*i*_), was estimated whereby *r*_*i*_ is the proportion of prey species *i* utilized and *P*_*i*_ is the proportion of prey species *i* available.

Inner reefs	Occupancy		Availability		Electivity index	
Octocoral spp.	*f*	*r*_*i*_	*f*	*P*_*i*_	(*e* = *r*_*i*_ − *P*_*i*_/*r*_*i*_ + *P*_*i*_)	Spp. utilization
*B. asbestinum *	0	0.000	25	0.019	−1.000	Total rejection
*G. ventalina *	10	0.156	370	0.280	−0.284	Total rejection
*P. americana *	33	0.516	496	0.375	0.158	Prop. abundance
*P. acerosa *	4	0.063	141	0.107	−0.259	Total rejection
*P. flexuosa *	8	0.125	154	0.117	0.033	Prop. abundance
*P. porosa *	8	0.125	101	0.076	0.244	Prop. abundance
*P. homomalla *	1	0.016	34	0.026	−0.238	Total rejection
Total	**64**	**1.0**	**1321**	**1.0**		

Mid-shelf reefs	Occupancy		Availability		Electivity index	
Octocoral spp.	*f*	*r*_*i*_	*f*	*P*_*i*_	(*e* = *r*_*i*_ − *P*_*i*_/*r*_*i*_ + *P*_*i*_)	Spp. utilization

*B. asbestinum *	5	0.125	58	0.040	0.515	Prop. abundance
*G. ventalina *	9	0.225	470	0.327	0.185	Prop. abundance
*P. americana *	10	0.250	255	0.177	0.171	Prop. abundance
*P. acerosa *	0	0.000	194	0.135	−1.000	Total rejection
*P. flexuosa *	10	0.250	250	0.174	0.179	Prop. abundance
*P. porosa *	3	0.075	182	0.127	−0.257	Total rejection
*P. homomalla *	3	0.075	28	0.019	0.596	Prop. abundance
Total	**40**	**1.0**	**1437**	**1.0**		

Shelf-Edge Reefs	Occupancy		Availability		Electivity index	
Octocoral spp.	*f*	*r*_*i*_	*f*	*P*_*i*_	(*e* = *r*_*i*_ − *P*_*i*_/*r*_*i*_ + *P*_*i*_)	Spp. utilization

*B. asbestinum *	11	0.105	47	0.059	0.280	Prop. abundance
*G. ventalina *	48	0.457	313	0.393	0.075	Prop. abundance
*P. americana *	18	0.171	76	0.095	0.286	Prop. abundance
*P. acerosa *	2	0.019	62	0.078	0.603	Prop. abundance
*P. flexuosa *	16	0.152	128	0.161	−0.029	Total rejection
*P. porosa *	10	0.095	142	0.178	−0.304	Total rejection
*P. homomalla *	0	0.000	28	0.035	−1.000	Total rejection
Total	**105**	**1.0**	**796**	**1.0**		

**Table 4:** Spearman rank correlations between the mean (%) PP and octocoral species density for the six most affected species across all reefs.

Octocoral spp.	*R*_*s*_	*P* value
*B. asbestinum *	0.09	0.80
^*^ *G. ventalina *	−0.94	0.02
*P. americana *	0.14	0.80
*P. acerosa *	0.09	0.92
*P. flexuosa *	−0.26	0.66
^*^ *P. porosa *	0.90	0.02

^*^Significant values (*P* < 0.05).

**Table 5:** Spearman rank correlations between the mean (%) PP and the pooled densities of all octocoral species within reefs.

Reef sites						
Spearman rank	Pelotas	Enrique	San Cristobal	Media Luna	Old Buoy	Weinberg
^*^ *R*_*s*_ (rho)	0.49	0.26	−0.73	−0.20	0.54	0.12
*P*-value	0.36	0.66	0.10	0.66	0.30	0.80

^*^ *R*_*s*_ coefficient = Spearman rank coefficient.

^*^Number of samples/correlation (*n* = 6 reefs).

**Table 6:** Mean percent (%), standard deviation, and standard error of (PP) (K-W; *α* = 0.05) for the six octocoral species showing the highest levels of PP within and across reefs.

Species	Reef	Number of transects	Number of colonies	Number of colonies (PP)	Mean %	SD	SE
*G. ventalina *	Pelotas	12	175	9	4.0%	0.04	0.01
*G. ventalina *	Enrique	12	195	9	3.8%	0.05	0.01
*G. ventalina *	San Cristobal	12	263	9	2.8%	0.04	0.01
*G. ventalina *	Media Luna	12	207	11	3.2%	0.05	0.01
*G. ventalina *	Old Buoy	12	152	32	20.7%	0.19	0.05
*G. ventalina *	Weinberg	12	161	25	16.3%	0.12	0.04

*P. acerosa *	Pelotas	12	58	3	17.4%	0.39	0.11
*P. acerosa *	Enrique	12	83	3	6.3%	0.19	0.05
*P. acerosa *	San Cristobal	12	118	1	2.8%	0.10	0.03
*P. acerosa *	Media Luna	12	76	1	4.2%	0.14	0.04
*P. acerosa *	Old Buoy	12	18	0	0.0%	0.00	0.00
*P. acerosa *	Weinberg	12	44	3	9.5%	0.19	0.06

*P. porosa *	Pelotas	12	25	6	13.7%	0.28	0.08
*P. porosa *	Enrique	12	76	15	23.6%	0.25	0.07
*P. porosa *	San Cristobal	12	106	14	15.7%	0.14	0.04
*P. porosa *	Media Luna	12	76	21	37.3%	0.35	0.10
*P. porosa *	Old Buoy	12	62	11	15.0%	0.22	0.06
*P. porosa *	Weinberg	12	80	9	19.5%	0.19	0.05

*P. flexuosa *	Pelotas	12	23	3	26.7%	0.39	0.11
*P. flexuosa *	Enrique	12	131	23	24.2%	0.32	0.09
*P. flexuosa *	San Cristobal	12	110	14	20.9%	0.28	0.08
*P. flexuosa *	Media Luna	12	140	12	26.0%	0.25	0.07
*P. flexuosa *	Old Buoy	12	48	8	16.7%	0.26	0.08
*P. flexuosa *	Weinberg	12	80	11	21.0%	0.20	0.06

*P. americana *	Pelotas	12	261	18	32.9%	0.32	0.09
*P. americana *	Enrique	12	235	14	6.7%	0.19	0.05
*P. americana *	San Cristobal	12	155	14	11.2%	0.17	0.05
*P. americana *	Media Luna	12	100	21	21.6%	0.32	0.09
*P. americana *	Old Buoy	12	45	10	22.9%	0.29	0.08
*P. americana *	Weinberg	12	31	10	14.0%	0.27	0.07

*B. asbestinum *	Pelotas	12	4	0	0.0%	0.0	0.0
*B. asbestinum *	Enrique	12	21	0	0.0%	0.0	0.0
*B. asbestinum *	San Cristobal	12	12	5	16.7%	0.31	0.09
*B. asbestinum *	Media Luna	12	46	0	5.6%	0.15	0.04
*B. asbestinum *	Old Buoy	12	10	2	5.6%	0.13	0.04
*B. asbestinum *	Weinberg	12	37	9	21.9%	0.28	0.08
